# Spontaneous resolution of atopic dermatitis incidental to participation in benralizumab clinical trial for severe, uncontrolled asthma: a case report

**DOI:** 10.1186/s13256-021-02663-2

**Published:** 2021-03-06

**Authors:** David N. Pham

**Affiliations:** Allianz Research Institute, 14120 Beach Blvd, Suite 101, Westminster, CA USA

**Keywords:** Atopy, Atopic dermatitis, Case report, Benralizumab, Eczema, Asthma, IL-5 receptor inhibitor, Eosinophilia

## Abstract

**Background:**

T cell-mediated eosinophilia is associated with numerous conditions—including atopic dermatitis, food allergies, and asthma—collectively known as the “atopic march.” Benralizumab is a recombinant, humanized, afucosylated monoclonal antibody directed against the ⍺ chain of the eosinophil cell surface receptor IL-5R. Benralizumab treatment causes near-complete depletion of circulating eosinophils and was approved in 2017 for add-on, maintenance treatment of severe asthma with an eosinophilic phenotype, based on the results of the CALIMA and SIROCCO pivotal trials. Benralizumab is not currently approved for the treatment of eosinophilic conditions besides asthma; however, during the CALIMA trial, spontaneous resolution of atopic dermatitis was observed in a patient, concurrent with reduction in her asthma symptoms.

**Case presentation:**

In January 2015, a 14-year-old Asian girl with severe, uncontrolled asthma was enrolled in CALIMA. The patient’s baseline eosinophil blood count was 1200 cells/μL, her pre-bronchodilator forced expiratory volume in 1 second (FEV_1_) was 1.9 L and FEV_1_/forced vital capacity (FVC) ratio was 71.4%, and her post-bronchodilator FEV_1_ was 3.2 L (FEV_1_/FVC of 115.9%). Her overall baseline asthma symptom score was 3.9 and her asthma exacerbation rate in the prior year was 4. She also displayed a pronounced, pruritic, chronic, inflammatory rash consistent with atopic dermatitis across her face. The investigator was blinded to the patient’s treatment group during treatment; however, her asthma symptoms diminished over the course of the study (FEV_1_ at 56 weeks, 3.01 L/110.5% (pre) and 3.25 L/119.3% (post); overall asthma symptom score 2.1; one influenza-associated exacerbation). Furthermore, her atopic dermatitis symptoms resolved spontaneously within the first 5 months of the study. After unblinding, the patient was confirmed to have been randomized to an active treatment arm, and her blood eosinophil count had dropped below the limit of detection after the first study dose.

**Conclusions:**

Given the potential shared mechanisms between eosinophilic asthma and atopic dermatitis, it is plausible that benralizumab-induced eosinopenia factored into the resolution of the patient’s atopic dermatitis. Further clinical studies are warranted to determine whether benralizumab or other drugs targeted against IL-5/IL-5R may be useful in managing multiple conditions associated with eosinophilia.

## Background

Eczema, or atopic dermatitis (AD), is a chronic, pruritic, inflammatory skin condition with a mechanism linked to deficiency of function in the skin barrier [1, 2]. Challenge by environmental allergens and pathogenic microbes precipitates a complex and exaggerated pro-inflammatory T cell response that includes interleukin-5 (IL-5) stimulation of eosinophil proliferation, activation, and subsequent overproduction of immunoglobulin E (IgE) [1, 2]. AD tends to precede or co-occur with other allergic diseases such as food allergies, severe eosinophilic asthma, and allergic rhinitis. It is increasingly understood that the physiologic mechanisms underlying these disorders overlap and may be part of a pathologic cascade [3].

The potential links between these allergic diseases point to a number of attractive therapeutic targets, including IgE antibodies, IL-5, and eosinophils, which are stimulated through the cell surface receptor, IL-5R [4–6]. Several pharmaceutical agents have been approved or are currently in development that are designed to disrupt the IL-5/IL-5R interaction in eosinophilic asthma, AD, or other allergic diseases [4]. However, it is not yet known whether agents developed against one indication will show efficacy against others within the constellation of atopic diseases.

Here, we report spontaneous resolution of AD in an adolescent patient participating in the phase 3 CALIMA pivotal trial, which evaluated the safety and efficacy of the anti-IL-5R mAb benralizumab as an add-on therapy for patients with severe, uncontrolled asthma and elevated blood eosinophil counts [7].

## Case presentation

In December 2013, a 14-year-old Asian girl (body mass index 22.34; never-smoker) was referred to our practice with severe, uncontrolled asthma, which was first diagnosed in 2009. At the time of referral, the patient was taking budesonide 160 μg and formoterol 4.5 μg, two puffs twice daily plus formoterol 18 μg daily. Her history was also significant for allergic rhinitis, AD, and IgE-confirmed allergies to an extensive panel of foods and to animal dander.

Her medications were adjusted to budesonide 160 μg and formoterol 4.5 μg taken as two puffs twice-daily, montelukast 5 mg daily, and salbutamol as needed; however, this regimen did not achieve satisfactory symptom control. In December 2014 she was screened for potential enrollment into the CALIMA clinical trial [7].

**Fig. 1:**
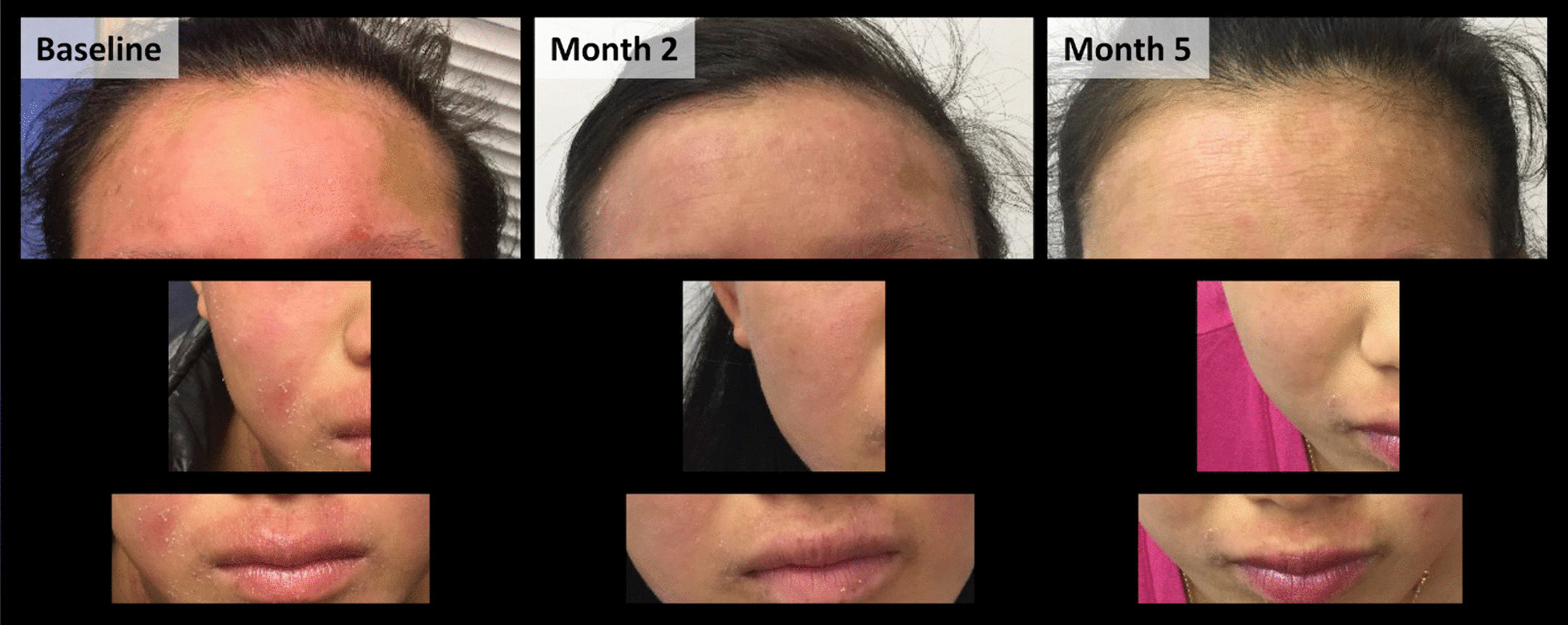
Progressive resolution of eczematous lesions in a patient participating in the CALIMA randomized clinical trial evaluating benralizumab as an add-on treatment for severe asthma with eosinophilic phenotype. The pictures were taken on the basis of clinical observation and without knowledge of the patient’s assigned dose group. After the trial was completed and unblinded, the patient was found to be in the treatment arm that received benralizumab 30 mg every 8 weeks, with the first three doses every 4 weeks, and in the primary analysis population of patients with at least 300 eosinophils/μL peripheral blood

At the enrollment visit in January 2015, the patient’s eosinophil blood count was 1200 cells/μL. Her pre-bronchodilator forced expiratory volume in 1 second (FEV_1_) was 1.9 L and FEV_1_/forced vital capacity (FVC) ratio was 71.4%, and her post-bronchodilator FEV_1_ was 3.2 L (FEV_1_/FVC of 115.9%). Her overall baseline asthma symptom score was 3.9. She had experienced four asthma exacerbations requiring treatment with systemic corticosteroids in the year prior to enrollment. Furthermore, a pronounced, pruritic, inflammatory rash consistent with AD was evident on the patient’s cheeks and forehead (Fig. 1, left panel). The patient and her guardian confirmed that the rash was chronic, and it was documented photographically as part of the patient record. Following completion of pre-enrollment screening and informed consent from her guardian, the patient was enrolled in the study and underwent the specified 4-week screening and run-in period [7]. She met all selection criteria and was randomized to blinded treatment assignment in January 2015. The patient completed a study visit every 4 weeks for 56 weeks for data collection and subcutaneous injection of her assigned study drug, and attended the 60-week final study visit. The patient was 100% compliant with the schedule and all study procedures. During the study, the patient continued using her background asthma controller medications, and was allowed to use albuterol two puffs, four times per day as needed as her rescue medication.

As CALIMA was a randomized, double-blind, placebo-controlled, parallel-group trial [7], it was not known to which arm the patient had been randomized and her eosinophil counts were redacted during the study and were unknown to the investigator or patient. However, during the course of treatment, the patient noted fewer asthma symptoms and reduced dyspnea during activities. Furthermore, a consistent and progressive resolution of her AD symptoms was observed and documented over 5 months (Fig. 1, middle and right panels). She experienced a single exacerbation requiring systemic corticosteroid treatment on day 363 of the study (December 2015), coincident with an influenza infection.

After the study was completed and unblinded, the patient was confirmed to have received 30 mg benralizumab every 8 weeks over the course of the 56-week treatment period, with the first three doses administered 4 weeks apart (the “Q8W” study arm). Her data were confirmed to have been included in the primary (intention-to-treat) analysis of patients with eosinophil count of at least 300 cells/μL.

At her 56-week study visit, the patient’s pre-bronchodilator FEV_1_ was 3.01 L (FEV_1_/FVC 110.5%), whereas her post-bronchodilator FEV_1_ was 3.3 L (FEV_1_/FVC 119.3%). Therefore, during the course of the study she gained 1.1 L in pre-bronchodilator FEV_1_. Furthermore, the patient’s overall asthma symptom score at 56 weeks was 2.1, a decrease of 1.8 points compared to baseline. For reference, in the CALIMA placebo group (*n* = 248), the least-squares mean change in pre-bronchodilator FEV_1_ was a 0.22-L increase, and the least-squares mean change in asthma symptom score was − 1.2 [7].

Furthermore, the patient’s blood eosinophil count dropped below the limit of detection by the 4-week visit (after one study dose) and remained there throughout the 56-week duration of the study.

## Discussion and conclusions

This case is notable in that the patient was enrolled in a clinical trial focused on a specific drug indication—severe, uncontrolled asthma with elevated blood eosinophil counts—but experienced dramatic resolution of symptoms for a different condition that may have a related mechanism of action. Evidence is increasing that the pathology of asthma, AD, and other diseases that are part of the so-called atopic march are a result of a self-reinforcing and progressive cascade of epithelial barrier dysfunction in the skin, gut mucosa, and respiratory mucosa, allowing increased penetration of allergens and microbes that elicit a systemic, T cell-mediated eosinophilic hyper-response [2, 3, 8]. Because this inappropriate eosinophilic response is characteristic of all of the atopic diseases, it is conceivable that treatments targeting the underlying T cell–eosinophil interaction could potentially show efficacy against more than one condition [2–6]. However this hypothesis must be investigated for each combination of drug and condition and the therapies currently on the market are, so far, limited to single indications.

Benralizumab is a recombinant, humanized, afucosylated monoclonal antibody (mAb) directed against the ⍺ chain of IL-5R, a receptor that is abundant on the surface of eosinophils and basophils and is essential to their function and survival [9, 10]. The engineered afucosylation enhances the receptor binding affinity of benralizumab as well as its antibody-dependent cell-mediated cytotoxicity function [9], resulting in near-total depletion of eosinophils from the circulation, and from the airways and lungs, and approximately 80% depletion of eosinophilic progenitors from the bone marrow [10, 11].

CALIMA was a randomized, double-blind, parallel-group, placebo-controlled, phase 3 clinical trial conducted at 303 sites in 11 countries [7]. Patients enrolled in the study were aged 12–75 years with severe asthma uncontrolled by medium- to high-dose inhaled corticosteroids (ICS) with long-acting β_2_-agonists (LABA) and a history of two or more exacerbations in the previous year. Patients were randomized in a 1:1:1 ratio to receive 56 weeks of treatment with benralizumab 30 mg subcutaneous every 4 weeks (Q4W) or every 8 weeks (Q8W; first three doses 4 weeks apart), or placebo. The study population was also stratified (2:1) by baseline blood eosinophil counts (at least 300 cells/μL versus less than 300 cells/μL). The primary endpoint was annual exacerbation rate ratio versus placebo for patients receiving high-dose ICS plus LABA with baseline blood eosinophils at least 300 cells/μL (intent-to-treat analysis). Secondary endpoints were FEV_1_ and total asthma symptom score.

Of 2505 patients enrolled and 1306 randomized, 728 patients were included in the primary analysis population. The study found that 56 weeks of add-on therapy with benralizumab at either dose level reduced annual exacerbation rates in patients with severe asthma and elevated baseline blood eosinophil counts by up to 36% compared with placebo (Q4W rate ratio 0.64 [95% CI 0.49–0.85], *p* = 0.0018, *n* = 241; and Q8W rate ratio 0.72 [95% CI 0.54–0.95], *p* = 0.0188, *n* = 239). Benralizumab also significantly improved pre-bronchodilator FEV_1_ in the Q4W and Q8W groups, and total asthma symptom score in the Q8W group. Furthermore, patients in both active dose groups experienced near-complete depletion of blood eosinophils by the 4-week sampling time point. The drug was well tolerated with few drug-related adverse events.

On the basis of the results of CALIMA and another pivotal trial, SIROCCO [7, 12], benralizumab was approved in 2017 by the US Food and Drug Administration as Fasenra^®^ for add-on maintenance treatment of patients with severe asthma, aged 12 years and older, with an eosinophilic phenotype [13]. The medication is not approved for treatment of other eosinophilic conditions and is not for relief of acute bronchospasm or status asthmaticus. Results from the first year of a long-term safety study (the BORA phase 3 extension trial) are consistent with efficacy and safety outcomes from the pivotal trials [14]. To date, benralizumab has not been evaluated clinically for its efficacy against AD, although a phase 2 study is being planned to further characterize its potential therapeutic role.

The asthma symptoms of the patient in this case responded to benralizumab in a manner consistent with the aggregate responses in patients in the Q8W arm of the CALIMA trial, with a reduced 56-week exacerbation rate, improved lung function, reduced asthma symptom score, and near-total depletion of blood eosinophils. The observed reduction in AD symptoms occurred concurrent with that response, as is evident in Fig. 1, with a substantial decrease in inflammation at 2 months compared with baseline, and skin healing at 5 months. It is noteworthy that the improvements in the patient’s AD symptoms occurred in the absence of any systemic steroid use for asthma exacerbation; the patient received systemic steroids only once during the study period, on day 363.

It is important to acknowledge that the findings reported here were incidental, observed during a case that was part of a large prospective clinical trial. A post hoc attempt to identify other patients in the study with similar findings would be practically infeasible and limited by unavoidable selection bias. However, the strength of the observed effect in this case suggests a potential area for investigation.

In conclusion, we observed spontaneous resolution of AD coincident with participation in a clinical trial for benralizumab to treat severe, uncontrolled, eosinophilic asthma. Given the plausible overlap in pathologic mechanisms between asthma and AD, further studies are warranted to determine whether benralizumab or other drugs targeted against IL-5/IL-5R may be efficacious in managing multiple allergic diseases.

## Data Availability

Not applicable.

## Abbreviations

ACQ-6: Asthma Control Questionnaire-6
AD: Atopic dermatitis
FEV_1_: Forced expiratory volume in 1 second
ICS: Inhaled corticosteroids
IgE: Immunoglobulin E
IL-5/IL-5R: Interleukin-5/interleukin-5 receptor
LABA: Long-acting β_2_-agonist
mAb: Monoclonal antibody
Q4W: CALIMA study group: benralizumab 30 mg every 4 weeks
Q8W: CALIMA study group: benralizumab 30 mg every 8 weeks, first three doses 4 weeks apart
